# Structure and evolution of a proviral locus of *Glyptapanteles indiensis *bracovirus

**DOI:** 10.1186/1471-2180-7-61

**Published:** 2007-06-26

**Authors:** Christopher A Desjardins, Dawn E Gundersen-Rindal, Jessica B Hostetler, Luke J Tallon, Roger W Fuester, Michael C Schatz, Monica J Pedroni, Douglas W Fadrosh, Brian J Haas, Bradley S Toms, Dan Chen, Vishvanath Nene

**Affiliations:** 1 The Institute for Genomic Research, a division of J. Craig Venter Institute, Rockville, Maryland, USA; 2 USDA-ARS Insect Biocontrol Laboratory, Beltsville, Maryland, USA; 3 USDA-ARS Beneficial Insect Introductions Research Laboratory, Newark, Delaware, USA; 4 Center for Bioinformatics and Computational Biology, University of Maryland, College Park, Maryland, USA; 5 Department of Biology, University of Rochester, Rochester, New York, USA

## Abstract

**Background:**

Bracoviruses (BVs), a group of double-stranded DNA viruses with segmented genomes, are mutualistic endosymbionts of parasitoid wasps. Virus particles are replication deficient and are produced only by female wasps from proviral sequences integrated into the wasp genome. Virus particles are injected along with eggs into caterpillar hosts, where viral gene expression facilitates parasitoid survival and therefore perpetuation of proviral DNA. Here we describe a 223 kbp region of *Glyptapanteles indiensis *genomic DNA which contains a part of the *G. indiensis *bracovirus (GiBV) proviral genome.

**Results:**

Eighteen of ~24 GiBV viral segment sequences are encoded by 7 non-overlapping sets of BAC clones, revealing that some proviral segment sequences are separated by long stretches of intervening DNA. Two overlapping BACs, which contain a locus of 8 tandemly arrayed proviral segments flanked on either side by ~35 kbp of non-packaged DNA, were sequenced and annotated. Structural and compositional analyses of this cluster revealed it exhibits a G+C and nucleotide composition distinct from the flanking DNA. By analyzing sequence polymorphisms in the 8 GiBV viral segment sequences, we found evidence for widespread selection acting on both protein-coding and non-coding DNA. Comparative analysis of viral and proviral segment sequences revealed a sequence motif involved in the excision of proviral genome segments which is highly conserved in two other bracoviruses.

**Conclusion:**

Contrary to current concepts of bracovirus proviral genome organization our results demonstrate that some but not all GiBV proviral segment sequences exist in a tandem array. Unexpectedly, non-coding DNA in the 8 proviral genome segments which typically occupies ~70% of BV viral genomes is under selection pressure suggesting it serves some function(s). We hypothesize that selection acting on GiBV proviral sequences maintains the genetic island-like nature of the cluster of proviral genome segments described herein. In contrast to large differences in the predicted gene composition of BV genomes, sequences that appear to mediate processes of viral segment formation, such as proviral segment excision and circularization, appear to be highly conserved, supporting the hypothesis of a single origin for BVs.

## Background

Much recent attention in genomics has focused on bacterial endosymbionts of insects, including the ubiquitous *Wolbachia *[1,2], the sap-feeder symbionts *Buchnera*, *Baumannia*, and *Sulcia *[3-5], and several others [6-8]. Many of these symbionts bring unique metabolic capabilities to their hosts, allowing these insects to flourish on diets which otherwise would be difficult to utilize. Less attention has been given to viral endosymbionts. Bracoviruses (BVs) and ichnoviruses (IVs) form subgroups of polydnaviruses (PDVs) that have evolved as obligate endosymbionts of braconid and ichneumonid endoparasitoid wasps, respectively, and appear to provide their primary hosts with pathogenic abilities [9]. Endoparasitoid wasps primarily parasitize other insects and usually kill the host organism they develop in. Most endoparasitoid wasps, including those that house PDVs, utilize a particularly difficult developmental strategy, known as koinobioncy, whereby the host continues to develop after it has been parasitized. Wasp eggs therefore begin development in a hostile environment in which they come under attack from the host's immune system. PDVs disrupt these responses.

Members of Polydnaviridae represent the only known viruses with segmented double-stranded DNA genomes [9]. They exist in two forms: as an asymptomatic proviral form integrated into the genome of male and female wasps [10-13], and as virions. Proviral DNA is amplified from wasp genomic DNA, and viral genome segments are excised, circularized, and packaged into virus particles only within specialized ovarian calyx cells of females [14-16]. Virions are released into the reproductive tract and do not appear to cause any ill effects. During oviposition, virions, along with wasp eggs and other factors, are injected into a secondary host, usually a caterpillar, where viral gene expression facilitates endoparasitoid survival by disrupting secondary host immunity, physiology, and development [17-19]. Additional wasp factors such as venom, ovarian proteins, and egg-associated teratocytes may contribute to parasitism success. Virus particles do not replicate within the secondary (or primary) host, yet viral-mediated pathology ensures perpetuation of the proviral form of the virus within the parasitoid life cycle.

PDVs are involved in a highly successful triad of mutualistic-parasitic relationships: it is estimated that there are over 30,000 wasp-PDV associations, with each wasp species exhibiting specific preferences in the host range they parasitize [20]. Drawing parallels from mitochondrial and bacterial endosymbiont genome evolution, some have hypothesized that PDVs are the product of reductive viral evolution [19,21]. Viral terminology is used to describe PDVs, although many unusual aspects of their biology have called into question this classification. Eukaryote-like genome properties and functional similarities between some PDV genes and components of wasp ovarian fluid have led to the suggestion that PDVs are not viruses at all, but rather represent genetic delivery vehicles that have acquired a virus-like packaging system and have evolved to transfer wasp parasitism genes to the lepidopteran host [22-24]. The evolutionary history of PDVs is further obscured by the hypothesis that, despite gross similarities in form and function, BVs and IVs have evolved independently [25,26]. Bracoviruses, however, are thought to be monophyletic, as all bracovirus-bearing wasps form a clade which originated ~74 million years ago [25].

To date six PDV viral genomes have been sequenced: CcBV and MdBV, BVs associated with the braconid wasps *Cotesia congregata *and *Microplitis demolitor*, respectively, and CsIV, HfIV, and TrIV, IVs associated with the ichneumonids *Campoletis sonorensis, Hyposoter fugitivus*, and *Tranosema rostrale *[24,27,28]. The sixth sequenced PDV, which is associated with the banchine ichneumonid *Glypta fumiferanae*, is hypothesized to form a third independent lineage of PDVs [29]. The packaged genomes of these viruses consist of between 15 and 105 circular segments and have aggregate sizes ranging from 189 to 568 Kbp. Unlike typical viruses only 17–30% of the viral genomes code for proteins, many genes are predicted to contain introns, and no genes code for obvious components of a DNA replication or transcription machinery. Thus, host enzymes may be utilized during construction of virus particles and/or viral genes may constitute part of proviral sequences which do not get packed into virus particles. In CsIV there is evidence for partitioning of genes encoding protein components of the virus particle between packaged and non-packaged genomic DNA [30,31], although no similar example has been shown for BVs. Compartmentalization of genes that are needed to maintain the PDV life cycle complicates study of virus biology and raises questions on the definition of sequences that constitute a PDV proviral genome.

While PDV viral genomes are better characterized, information on proviral genomes is limited. Studies on the location of proviral genome segment sequences in CsIV suggest that IV proviral genomes are integrated at multiple loci in the wasp genome [32]. By contrast, it is thought that BV proviral genome segments are tandemly arrayed in a single locus and separated by short intervening sequences [12,33-35]. The latter hypothesis is based on studies of CcBV and CiBV in which proviral genome segments were flanked, at least on one end, by a different proviral genome segment [12,33] and a fluorescent in situ hybridization mapping study in which probes from three different CcBV viral genome segments hybridized to the same region of a single wasp chromosome [35].

The current model for production of BV viral segment sequences is that one or more large precursor molecules encompassing multiple proviral viral genome segments are excised from genomic DNA and amplified, and this DNA forms the substrate from which viral segments are excised [34,36,37]. According to studies of CcBV and CiBV (BV associated with *Chelonus inanitus*), all amplification of BV DNA occurs at the level of the precursor molecule–no amplification occurs following excision of viral genome segments [34,36,37]. The DNA sequence at the segmental boundaries of a limited number of proviral genome segments of CsIV, CiBV and CcBV have been studied [11,33,38-40], and, in each, a direct DNA sequence repeat occurs at the boundaries. Proviral genome segment sequences are excised from the precursor molecules at these repeats, possibly via conservative site-specific recombination, and a single copy of the repeat is retained within the circularized viral segment [12]. Additionally, genome segments are packaged into virus particles in different abundances [28,33,40,41]. Recent semi-quantitative studies have shown large differences in copy number in both viral (MdBV and CiBV) and proviral (CiBV only) forms of segments [40,42]. The details of this phenomenon and its relationship to amplification and excision are unknown.

Here we describe the analyses of a 223 kbp section of genomic DNA from the braconid *Glyptapanteles indiensis *which parasitizes gypsy moth. This region contains 8 proviral genome segments of *G. indiensis *Bracovirus (GiBV). Our data provide new insight into BV proviral genome structure, as not all GiBV viral genome segment sequences are linked in a single tandem array in the wasp genome. Conserved DNA sequences identified at the junctions of GiBV proviral genome segment sequences and in GiBV, CcBV and MdBV viral segments suggest that sequence motifs governing segment excision are highly conserved across bracoviruses. Analyses of GiBV viral segment sequence polymorphism data indicate that widespread selection acts on non-coding DNA, suggesting additional functional motifs or non-coding RNAs are present in the GiBV viral genome. Finally, there is a marked difference in nucleotide composition between proviral segment sequences and flanking DNA that is not packaged into virus particles.

## Results

### Partial sequence characterization of GiBV viral DNA

Viral DNA was subjected to whole genome shotgun sequencing using purified virus pooled from the calyx fluid of ~400 female wasps from an outbred population. As judged by sizing on agarose gels, the GiBV viral genome was expected to contain 13 segments with a genome size of ~250 kbp [41]. However, assembly of our preliminary sequence data indicate an aggregate genome size of ~490 kbp and ~24 different segments. Many segments are of similar sizes and would have co-migrated on agarose gels. A high frequency of single nucleotide polymorphism (SNP) (~1/70 bp) and insertions and deletions (indels) in the DNA of the viral population that was sampled complicated the closure phase of the sequencing project. Nevertheless, 19 of the 24 preliminary viral genome segment sequences were of sufficient quality to allow development of segment-specific PCR primers (data not shown). These primers were used to determine the proviral genome segment composition of BAC clones that hybridized with ^32^P-labeled GiBV viral DNA. Priority was given to closing sequence and physical gaps in 8 viral genome segments that were encoded by two overlapping BAC clones (see below). A consensus sequence was generated for each viral genome segment (see Materials and Methods), and the resulting sequences, which varied in length from 10 to 26 kbp, were deposited in GenBank (EF051505–EF051512). Individual sequence reads were deposited in the NCBI Trace Archive (1472627677-1472629890).

### Identification of BAC clones containing GiBV proviral DNA

Radioactive probes derived from total GiBV viral DNA hybridized at varying intensity to 127 clones from a BAC library of 9,216 clones made from the larvae of *G. indiensis*. Nineteen viral genome segment-specific PCRs were used to genotype 60 BAC clones to determine the proviral genome segment composition. These BAC clones segregated into 7 sets that contained non-overlapping profiles of viral genome segments (Table 1). Each set contained 1 to 7 proviral genome segments, and in total 17 of the 19 proviral genome segments were identified. Additionally, a sub-set of 30 BAC clones were fingerprinted using *Eco*RI and the resulting restriction enzyme patterns were used to place the BAC clones into overlapping contigs. This method of clustering was consistent with the results of the segment-specific PCRs (data not shown).

**Table 1:** Proviral genome segment composition of 60 GiBV BAC clones.

**Genome Segment Set**	**Number of Genome Segments**	**Number of positive BACs**	**Number of BACs tested**
1	7	7	20
2	4	5	30
3	2	1	60
4	1	3	30
5	1	1	60
6	1	3	60
7	1	1	60

Non-overlapping sets of proviral genome segments found in BAC clones, arbitrarily designated as set 1–7, are shown in column 1. The second column shows the number of proviral genome segments identified in each set. The third column shows the number of BACs which tested positive for that set, and the fourth column shows the number of BACs that were tested for that set. Some segment sets were tested for on less than 60 BAC clones, as once multiple clones were identified for a set of proviral genome segments, the primer pairs representing those sets of segments were removed from PCR experiments to reduce the number of PCRs needed to identify the entire proviral genome.

### Structure and composition of GiBV proviral locus 1

Two overlapping BAC clones that appeared to code for a cluster of 7 proviral genome segments were selected for sequencing. BAC clones 18I8 and 20D14 were 120,708 kbp and 116,222 kbp in length, respectively, and overlapped by 14,273 bp. The region of sequence overlap contained 53 SNPs, indicating the BAC clones were derived from different individuals from a population of *G. indiensis*. A contiguous DNA sequence was generated by fusing positions 1–109,055 of clone 18I8 with positions 2,560–116,222 of clone 20D14, resulting in a region spanning 222,657 bp. The annotated DNA sequence was deposited in GenBank (AC191960).

**Figure 1:**
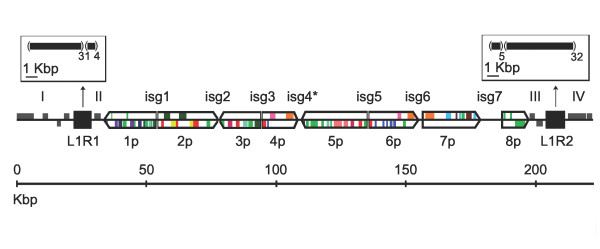
**Structural organization of GiBV proviral locus 1**. Proviral genome segments are labeled 1p-8p, with the square and pointed ends representing the 5' and 3' ends, respectively, relative to the putative excision motif. Inter-segmental regions are labeled isg1-isg7, and sequence regions outside the proviral genome segment sequences are labeled I-IV. The flanking tandem repeat regions (solid black squares) are labeled L1R1 and L1R2, and their structure is shown in the open boxes as black boxes in parentheses followed by the copy number of repeat as a subscript. The 2 BAC sequences were joined in isg4 (*) allowing the entirety of each proviral segment sequence to originate from a single BAC clone. Colored boxes represent genes; grey boxes are non-packaged genes, light green boxes are hypothetical proteins without gene family assignment, and the remaining colors represent different gene families.

The coordinates of the 7 proviral genome segment sequences in this region were determined by aligning viral genome segment sequences to it. A search of the BAC sequences against the entire assembly of viral genome segment shotgun sequence data led to the identification and closure of an extra viral genome segment sequence. This assembly was not of high enough quality for primer design during the BAC clone screening phase. Thus, a cluster of 8 proviral genome segments labeled 1p to 8p separated by 7 inter-segmental regions (isg1 to isg7) that vary in length from 122 bp to 8.4 kbp occupies ~163 kbp of DNA which we call GiBV proviral locus 1. Interestingly, the 34 kbp and 25 kbp region of DNA that flank locus 1 contain a 6–7 kbp section of DNA (L1R1 and L1R2) consisting primarily of non-coding tandem DNA sequence repeats (Figure 1, Table 2).

**Table 2:** Features of the regions of GiBV proviral locus 1

**Region**	**Coordinates**	**Size (bp)**	**% G+C (c/n-c)**	**% Coding**	**Predicted genes**
I	1 – 23133	23133	31 (47/27)	22	4
L1R1	23134 – 29250	6117	38	0	0
II	29251 – 34177	4927	35 (42/32)	36	1
1p	34178 – 54542	20365	37 (38/36)	38	14
isg1	54543 – 54769	227	30	0	0
2p	54770 – 78277	23508	36 (44/34)	25	8
isg2	78278 – 78394	117	29	0	0
3p	78395 – 94733	16339	37 (41/35)	35	6
isg3	94734 – 94903	170	26	0	0
4p	94904 – 108614	13711	36 (41/31)	42	4
isg4	108615 – 110126	1512	27	0	0
5p	110127 – 135963	25837	37 (41/34)	41	11
isg5	135964 – 136085	122	28	0	0
6p	136086 – 155462	19377	37 (37/37)	33	9
isg6	155463 – 156602	1140	29	0	0
7p	156603 – 179005	22403	36 (41/32)	35	7
isg7	179006 – 187374	8369	25	0	0
8p	187375 – 197431	10057	38 (42/34)	47	3
III	197432 – 204112	6681	33 (43/28)	33	2
L1R2	204113 – 211240	7128	37	0	0
IV	211241 – 222657	11417	30 (43/27)	22	2

Coordinates are with respect to the sequence of the entire locus. The % G+C column is divided into coding (c) and non-coding (n-c) for regions predicted to encode genes.

**Figure 2:**
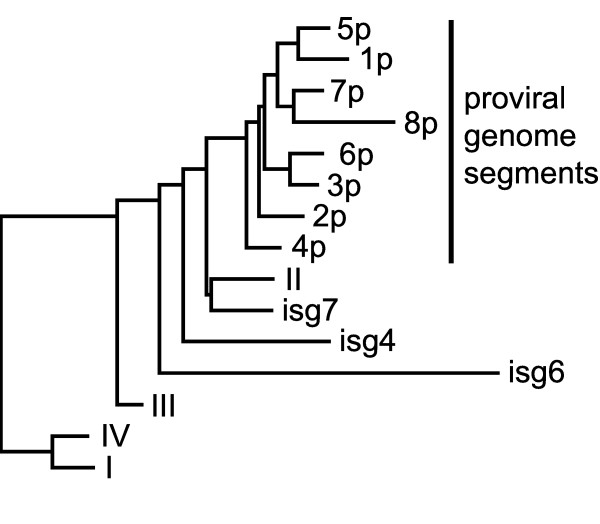
**Neighbor-joining clustering of the regions of proviral locus 1 based on relative dinucleotide frequencies**. All proviral genome segments (1p-8p) group together, as do the regions outside the flanking repeats (I and IV). The scale represents the normalized Euclidean distance between regions. Regions < 500 bp (isg1–3, 5) and the flanking repeats (L1R1 and L1R2) were excluded from the analysis, as they have skewed dinucleotide frequencies.

A variety of nucleotide compositional differences exist between the flanking regions I-IV, inter-segmental regions, and proviral genome segments. The latter sequences and L1R1/L1R2 have the highest average G+C content (37%), followed by the flanking regions (32%) while the inter-segmental regions have the lowest G+C content (26%). The difference in G+C content between coding and non-coding DNA is greater in flanking regions I-IV (44% vs. 28%) than in proviral genome segment sequences (41% vs. 34%) (Table 2). Relative dinucleotide frequencies which correct for background G+C composition were calculated for each region > 500 bp in length, except L1R1 and L1R2 as tandemly repetitive sequences have highly biased dinucleotide frequencies. Neighbor-joining clustering of the distances derived from these data (Figure 2) revealed that all of the proviral genome segments cluster together and have a highly similar dinucleotide composition, which is distinct from flanking DNA. Regions I and IV clustered together and the most distantly from proviral genome segments, whereas regions II and III and the inter-segmental regions clustered between the proviral genome segments and regions I and IV.

### A conserved DNA sequence motif exists at proviral genome segment junctions

**Figure 3:**
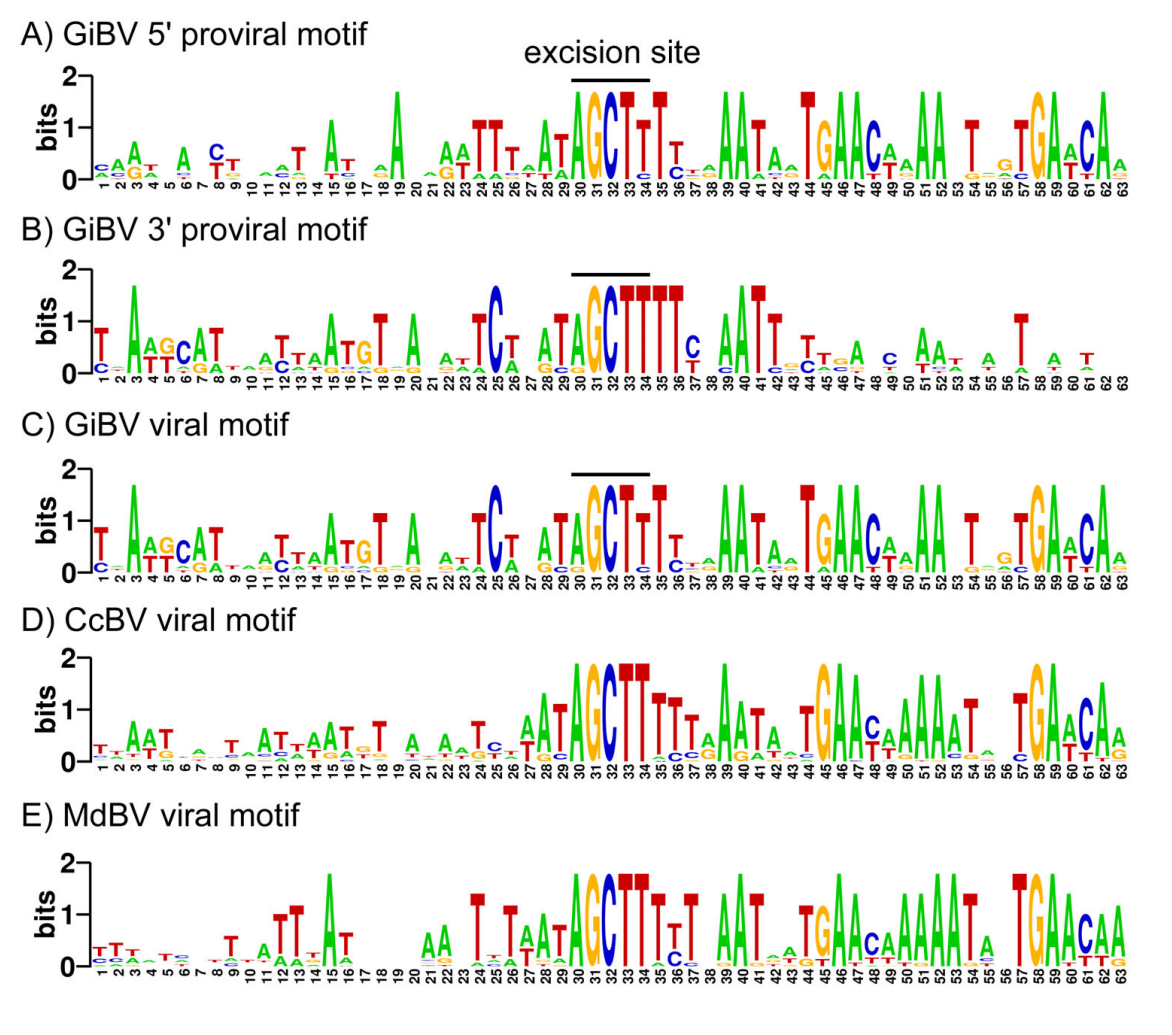
**Nucleotide conservation extended 30 bp in both directions around the GCT excision site**. A) 5' motif of proviral genome segments in GiBV proviral locus 1, in which sequence to the left of the motif represents inter-segmental sequences and sequence to the right of the motif represents proviral genome segment sequences. B) 3' motif of proviral genome segments GiBV proviral locus 1, in which the positions of inter-segmental and proviral genome segment sequences are reversed with respect to A). C) Extended motif from the 8 viral genome segments in proviral locus 1. D) Extended motif from all 30 CcBV viral genome segments. E) Extended motif from 13 of 15 MdBV viral genome segments.

Visual examination of the DNA sequence at the junctions between GiBV proviral genome segments and inter-segmental regions led to the identification of a 6 bp direct sequence repeat (AGCTTT), which is perfectly conserved at 14 of the 16 junctions and has one nucleotide substitution at the remaining 2 junctions. Because this repeat is encoded on the top DNA strand for 3 proviral genome segments (1p, 3, p, and 5p) and the bottom DNA strand for the remaining 5 proviral genome segments (2p, 4p, 6p, 7p, and 8p), the 5' and 3' boundaries of a proviral genome segment were defined as the first and second copy of the AGCTTT repeat relative to the sequence depicted in Figure 1. The 16 junction sequences were separated into 5' and 3' boundaries and searched using MEME, a motif discovery tool. An extended sequence motif centered on the AGCTTT repeat was identified in each group of sequences. The 5' and 3' motifs are different to each other and the 5' motif is more conserved than the 3' motif. Conservation of both motifs was greater and longer on the segmental side of the excision site than on the inter-segmental side (Figure 3).

MEME analysis of the 8 GiBV viral genome segment sequences revealed the presence of a single copy of the AGCTTT repeat surrounded by a recombined motif from the 5' and 3' motifs (Figure 3). By comparing proviral and viral genome segment sequences, it was determined that the two nucleotide polymorphisms present in the AGCTTT repeat of the proviral genome segment sequences appeared in the single copy of the repeat in viral genome segment sequences. Specifically, the 5' repeat of segment 5p has a substitution at the fifth position while the 3' repeat of segment 3p has a substitution at the first position and both changes occur in the corresponding viral segment.

MEME was also used to search the complete CcBV and MdBV viral genomes, and the 5 available viral genome segments of CiBV. A sequence motif highly similar to the recombined GiBV segment motif was found in all 30 CcBV viral genome segments and 13 out of 15 MdBV viral genome segments (Figure 3). No similar motif was found in CiBV, although described CiBV exision sites show conservation of varying degrees to the AGCTTT repeat [33,40].

### Annotation of proviral locus 1 and flanking DNA

Two previously described GiBV cDNAs (p325 and p494) expressed in infected gypsy moth larvae [41] which encode hypothetical proteins map to multiple genes in proviral locus 1. These cDNAs provide direct evidence for the presence of 1 and 2 introns in the p325 and p494 gene families, respectively; p494 maps to 2 genes in proviral genome segment 2p, while p325 maps to 1 gene of proviral genome segment 3p, 4p, and 5p. The shortest and longest intron was 83 bp and 591 bp in length, respectively. Four variations of *ab initio *gene modeling programs were tested for their ability to recover the correct intron-exon structure of these 5 genes. A combination of Softberry's FGENESH trained on the honey bee (*Apis mellifera*) and the Beijing Genome Institute's BGF trained on the silkmoth (*Bombyx mori*) were most accurate and these programs, in addition to protein alignments generated with the AAT package [43] were used to predict protein-coding gene models (see Materials and Methods).

A total of 62 protein-coding genes were predicted to be encoded within the 8 proviral genome segments (Table 2 and 3). As judged by sequence similarity using BLASTP, 47 genes have homologs in CcBV, but only 3 genes, all of which are members of hypothetical family 4, have homologs in MdBV. A TBLASTN analysis of the predicted GiBV proteins against the MdBV and CcBV genomes showed no additional similarity to MdBV. However, of the 15 proteins which did not show BLASTP similarity to CcBV, 5 showed similarity to translated CcBV sequences, suggesting homologs of these genes may exist in CcBV but were not previously predicted. The 10 remaining GiBV genes which do not have homologs in CcBV encode novel hypothetical proteins.

**Table 3:** Annotation of proviral locus 1

**Gene identifier**	**Region**	**Size**	**Introns**	**Sigs**	**Product**	**Family**	**dN/dS**
GIP_L1_00010	I	500	4		FL(2)D protein		
GIP_L1_00020	I	369	2		Trans-2-enoyl-CoA reductase		
GIP_L1_00030	I	240	2		oxidored-nitro domain-like protein		
GIP_L1_00040	I	562	3		hypothetical protein		
GIP_L1_00050	II	599	4	s	5' nucleotidase		
GIP_L1_00060	1p	165	1	s, t	hypothetical protein	3	*
GIP_L1_00070	1p	98	1		lectin-like protein		*
GIP_L1_00080	1p	210	1	s, t	conserved hypothetical protein	3	0.29
GIP_L1_00090	1p	266	1	s, t	conserved hypothetical protein		0.51
GIP_L1_00100	1p	304	1	s	CrV1-like protein	5	0.77
GIP_L1_00110	1p	161	1	s	Lectin C-type domain		0.54
GIP_L1_00120	1p	138	1		conserved hypothetical protein	3	0.81
GIP_L1_00130	1p	133	0	s	Cystatin domain		0.38
GIP_L1_00140	1p	341	1	s	CrV1-like protein	5	0.51
GIP_L1_00150	1p	195	1	s	hypothetical protein	5	1.04
GIP_L1_00160	1p	104	1		hypothetical protein		*
GIP_L1_00170	1p	219	1	s, g	conserved hypothetical protein	7	*
GIP_L1_00180	1p	78	0	s	hypothetical protein		*
GIP_L1_00190	1p	198	1		hypothetical protein	10	*
GIP_L1_00200	2p	143	1	s	conserved hypothetical protein	1	u
GIP_L1_00210	2p	494	2	s	P494 protein	8	*
GIP_L1_00220	2p	97	1	s	hypothetical protein	9	*
GIP_L1_00230	2p	147	1	s	conserved hypothetical protein	1	*
GIP_L1_00240	2p	582	2	s	P494 protein	8	*
GIP_L1_00250	2p	88	1		hypothetical protein	9	*
GIP_L1_00260	2p	147	1	s	conserved hypothetical protein	1	*
GIP_L1_00270	2p	253	1	s	conserved hypothetical protein		*
GIP_L1_00280	3p	320	1	s	conserved hypothetical protein		*
GIP_L1_00290	3p	354	1	s	conserved hypothetical protein	12	0.09
GIP_L1_00300	3p	340	1	s, g	P325 protein	1	0.56
GIP_L1_00310	3p	226	1	s	conserved hypothetical protein	7	0.56
GIP_L1_00320	3p	241	1	s, g	hypothetical protein		0.29
GIP_L1_00330	3p	444	1	s	hypothetical protein	10	2.12
GIP_L1_00340	4p	337	1	s, g	P325 protein	1	0.37
GIP_L1_00350	4p	106	1	s	conserved hypothetical protein	2	*
GIP_L1_00360	4p	597	2	s	Ribonuclease T2 domain	11	1.96
GIP_L1_00370	4p	898	2		conserved hypothetical protein	4	0.64
GIP_L1_00380	5p	166	1	s, t	hypothetical protein	3	*
GIP_L1_00390	5p	171	1	s	hypothetical protein		0.4
GIP_L1_00400	5p	430	1	s, g	conserved hypothetical protein		0.55
GIP_L1_00410	5p	247	1	s	conserved hypothetical protein		0.06
GIP_L1_00420	5p	215	1	s	conserved hypothetical protein	7	0.31
GIP_L1_00430	5p	108	1	s, t	hypothetical protein		*
GIP_L1_00440	5p	767	1	s	lipoprotein-like protein	14	0.5
GIP_L1_00450	5p	581	0	s	conserved hypothetical protein	14	0.53
GIP_L1_00460	5p	348	1	s	conserved hypothetical protein	12	0.55
GIP_L1_00470	5p	304	1	s	P325 protein	1	2.21
GIP_L1_00480	5p	170	1	s	conserved hypothetical protein		*
GIP_L1_00490	6p	279	1	g	P325-like protein	1	0.35
GIP_L1_00500	6p	109	1	s	conserved hypothetical protein	2	0.18
GIP_L1_00510	6p	140	1	s	conserved hypothetical protein	2	*
GIP_L1_00520	6p	100	1	s	conserved hypothetical protein	2	0.57
GIP_L1_00530	6p	101	1	s	conserved hypothetical protein	2	0.21
GIP_L1_00540	6p	106	1	s	conserved hypothetical protein	2	*
GIP_L1_00550	6p	293	1		Ribonuclease T2 domain	11	0.51
GIP_L1_00560	6p	118	1		hypothetical protein		*
GIP_L1_00570	6p	896	2		conserved hypothetical protein	4	0.54
GIP_L1_00580	7p	1066	2		conserved hypothetical protein	4	0.57
GIP_L1_00590	7p	478	2	s	conserved hypothetical protein	6	0.75
GIP_L1_00600	7p	119	1	s	conserved hypothetical protein	13	*
GIP_L1_00610	7p	109	1	s	conserved hypothetical protein	6	0.59
GIP_L1_00620	7p	218	1		conserved hypothetical protein		0.74
GIP_L1_00630	7p	496	1	s	conserved hypothetical protein	13	0.58
GIP_L1_00640	7p	127	2	s	conserved hypothetical protein	6	0.57
GIP_L1_00650	8p	253	1	s, t	EP1-like protein		6.01
GIP_L1_00660	8p	177	1	s, g	conserved hypothetical protein		0.92
GIP_L1_00670	8p	1132	1	s	dentin-like protein		0.72
GIP_L1_00680	III	599	1	s	hypothetical protein		
GIP_L1_00690	III	130	1		hypothetical protein		
GIP_L1_00700	IV	480	6		N-myristoyltransferase		
GIP_L1_00710	IV	326	3		Hyaluronidase		

Gene identifier indicates the Genbank locus tag for each predicted gene. Region is the location of genes according to the delineations in Table 2. Sizes of the genes are given in amino acids. Signatures (Sigs) include "s" signal peptide, "t" trans-membrane domain, and "g" potential glycosylphosphatidylinisotol anchor. Family indicates the gene family to which the predicted gene belongs, if any. dN/dS ratios are given when applicable, and an "*" represents insufficient data to calculate a ratio, while a "u" represents a mathematically undefined ratio.

Only 10 of the 62 predicted GiBV genes in the locus were assigned a potential function, namely C-type lectins and proteins containing a cystatin or ribonuclease T2 domain (Table 3). Surprisingly, 50 genes were predicted to access the secretory pathway as they contained a signal peptide at the N-terminus. Of these 6 genes were predicted to have trans-membrane domains, and 6 genes were predicted to have potential glycosylphosphatidylinisotol anchors. Only 3 proviral genome segment genes were not predicted to contain introns and the remaining genes contain either 1 or 2 introns. A protein domain-based clustering pipeline placed 43 of the 62 proteins into 14 gene families (see Methods and Table 3). The distribution of members of these gene families was generally not restricted to specific proviral genome segments–8 gene families, including all families with 4 members or more, were located on at least 2 non-adjoining proviral genome segments.

Regions L1R1 and L1R2 and the inter-segmental regions were not predicted to contain protein-coding genes, nor did these sequences produce any significant matches when tested against the GenBank non-redundant protein database using BLASTX (E = e-10). On the other hand, regions I to IV were predicted to encode 9 genes and potential function was assigned to 6 of them (Table 3). These genes had a top blast hit to genes from *Apis mellifera *(BLASTP, E < e-45), including the 5'-nucleotidase, trans-2-enoyl-CoA reductase, hyaluronidase, N-myristoyltransferase, and 1 hypothetical protein. By contrast, none of the genes encoded by the proviral genome segments had any sequence similarity to *A. mellifera *(BLASTP, E = e-10), other than proteins with conserved domains encoded in a large number of genomes (e.g., the C-type lectin domain). Four of 6 genes are encoded on *A. mellifera *chromosome 14, although only the honey bee hyaluronidase and N-myristoyltransferase genes were located in close proximity to each other.

### Analysis of sequence polymorphisms in GiBV viral genome segment sequences

Proviral genome segments in locus 1 share 99.5–99.9% sequence identity with their homologous viral genome segment sequence. The distribution of 2,159 SNPs in the 8 GiBV viral genome segment sequences relative to the corresponding proviral genome segment sequence is shown in Table 4. Viral genome segment 2 showed a low frequency of polymorphisms, averaging ~5 SNPs/kbp, while the remaining segments had an average SNP density of ~16 SNPs/kbp. The majority of genome segments showed no significant correlation between sequence coverage and SNP density (Table 4), with the exception of segment 1, which showed a slight correlation (R^2 ^= 0.25, p < 0.05). All SNPs were placed in one of three classes: non-coding, synonymous, and non-synonymous. As expected, there was a significantly higher SNP density in synonymous sites than non-synonymous sites (χ^2^_1 df _= 37.3, p < 0.01). However, there was also a higher SNP density in synonymous sites relative to non-coding sites (χ^2^_1 df _= 38.2, p < 0.01), and no difference in SNP density between non-coding and non-synonymous sites (χ^2^_1 df _= 1.8, p > 0.05).

**Table 4:** Single Nucleotide Polymorphisms (SNPs) in the viral genome segment sequences

			**GiVB genome segment**						
	**1**	**2**	**3**	**4**	**5**	**6**	**7**	**8**	**Total**

SNPs	351	107	270	166	216	354	421	174	2159
per Kbp	17.53	4.55	16.52	12.18	12.27	18.32	18.79	17.40	
Non-Coding	232	91	149	74	195	239	269	102	1351
Coding	119	16	121	92	121	115	152	72	808
Synonymous	36	3	37	27	44	42	46	15	250
Non-synonymous	83	13	84	65	77	73	106	57	558
Coverage	10.1	11.5	9.8	9.8	16.3	10.9	10.3	5.2	
R^2^	0.25*	< 0.01	0.14	< 0.01	< 0.01	0.05	0.02	0.08	

*p < 0.05

Coverage indicates average sequence coverage across the viral genome segment in the whole genome shotgun, and R^2 ^represents the correlation between the number of SNPs and sequence coverage. Only viral genome segment 1 showed a significant (p < 0.05) correlation between SNP density and coverage.

**Figure 4:**
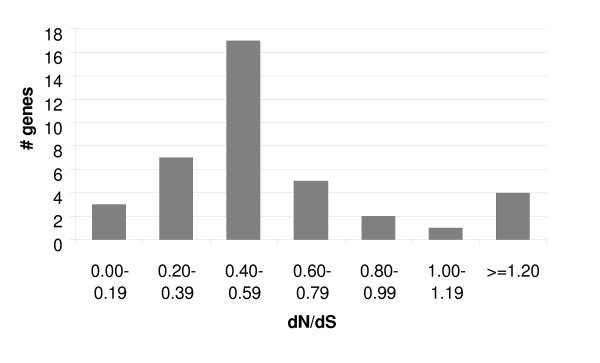
Histogram of dN/dS ratios of 39 genes in the viral genome segments.

The number of SNPs per gene ranged from 0 to 68, and dN/dS ratios were calculated for the 39 out of 62 genes that contained 5 or more SNPs (Table 3). Most of these genes appear to be under purifying selection and 32 of 39 genes had dN/dS ratio < 0.8 with a majority of the ratios falling in the range of 0.40–0.59 (Figure 4). Three genes appear to be evolving neutrally (dN/dS = 0.8–1.2) and code for 2 hypothetical proteins and 1 member of gene family 3. Four genes had a dN/dS > 1.9, including 1 member each of gene families 1, 10, and 11 (the ribonuclease T2 domain) and an EP1-like protein. No correlation was found between dN/dS ratios and specific genome segments or gene families–most segments and gene families contained genes under different degrees of selection.

## Discussion

### Not all GiBV proviral genome segments occur in a tandem array

Prior to this study, it was believed that bracovirus proviral genome segments were closely linked in a tandem array in the wasp genome with short stretches of intervening DNA separating them [12,33-35]. Our study indicates that this is not the case for GiBV. While some GiBV proviral genome segment sequences are clustered in tandem arrays others occur in isolation as singletons. This conclusion is supported by the segregation of BAC clones coding for 18 of ~24 proviral genome segments into 7 non-overlapping sets of clones via viral genome segment-specific PCRs (Table 1), and preliminary BAC shotgun sequence data support the typing data (not shown). Furthermore, although we describe a tandem array of proviral genome segments in this paper at GiBV proviral locus 1, the array codes for only 8 proviral genome segment sequences and this cluster is flanked by at least 34 kbp and 25 kbp of DNA (Figure 1) that is not packaged into GiBV virions. It remains to be determined whether the 7 loci encoding GiBV proviral segment sequences are linked on the same chromosome as a macrolocus but with longer stretches of intervening DNA between them, or whether they are dispersed across more than one chromosome. Although the former scenario remains compatible with a study of *C. congregata *where probes from 3 different viral genome segments bound to the same location on *C. congregata *chromosome 5 [35], the structural organization of BV proviral genome segment sequences appears to be more complex than previously hypothesized.

It is reasonable to propose that the inter-segmental regions in GiBV proviral locus 1 should be classified as part of the GiBV proviral genome. However, to what extent the proviral genome extends into flanking DNA is less easily determined. BV viral genome segments are thought to be excised from the amplified products of one or more large precursor molecules, and there is no evidence for post-excision amplification of segments [34,36,37]. Thus copy number studies of regions immediately flanking GiBV proviral locus 1 and other loci containing proviral genome segment sequences at the time of viral genome segment formation could be used as a surrogate marker for identifying potential components of the GiBV proviral genome.

### Gene content of proviral locus 1 and flanking regions

Due to the limited transcriptional data available for BVs, there is substantial disagreement on the structural complexity of BV genes, particularly with regards to the percentage of PDV genes that contain introns. While Espagne *et al *[24] predicted that 69% of CcBV proteins contain introns, Webb *et al *[28] re-annotated the CcBV genome and predicted only 6.8% of CcBV genes contain introns–a ten-fold difference in intron content. In GiBV proviral locus 1, using a combination of Hymenoptera- and Lepidoptera-trained gene prediction programs (see Methods), we predicted that 81% of the 63 genes contain introns. Sequence data from 2 cDNAs derived from genes in proviral locus 1 suggests that the 7 introns predicted for 5 members of the 2 gene families are real and not artifacts of improper gene modeling. However, this number is probably not reflective of the entire GiBV genome, as PTP and ankyrin genes usually do not contain introns [44-46] and generally comprise a large percentage of BV genes (21% and 41% of predicted CcBV and MdBV genes, respectively), but are not present in GiBV proviral locus 1. Regardless, the accuracy of most predicted gene models awaits experimental verification. While the presence of introns may be unusual for virus genes, some DNA viruses which replicate in the host cell nucleus encode genes with introns (e.g., adenoviruses [47]).

GiBV genes in proviral locus 1 predicted to contain introns have an extremely simple intron-exon structure compared to often complex higher eukaryotic genes, and generally contain a single short exon followed by a long exon encoding the remainder of the protein. Remarkably, 80% of the genes at this locus, including the p494 and p325 gene families which are transcribed in infected gypsy moth larvae, are predicted to encode a secretion signal peptide within the first exon. Secretion of some proteins may compensate for differences in the abundance of segment sequences in virions. Since it is unclear whether the entirety of the GiBV genome is packaged into a single virion [41], secretion of a large number of proteins may be necessary for properly delivery of these proteins. Attempts to functionally annotate the 62 predicted genes in the 8 GiBV proviral genome segment sequences identified the presence of a C-type lectin [48], CrV1-like proteins [49], and a number of conserved hypothetical proteins encoded by other PDV genomes [19,24,26,50]. Most of the genes in locus 1 were predicted to have homologs in CcBV, while only gene family 4 showed homology to a gene on MdBV segment B. Although the function of this gene family is unknown, it is the only gene family in GiBV proviral locus 1 for which none of the members are predicted to contain signal peptides.

The placement of 43 GiBV genes into 14 gene families suggests that extensive duplication of genes has occurred within proviral locus 1. Typically, gene duplications are thought to result in relaxation of the selection on the duplicated gene, allowing it to acquire a new function. However, the majority of genes in proviral locus 1, even multiple members of the same gene family, appear to be under purifying selection (Figure 4). This implies that members of gene families are, for the most part, not free to acquire entirely new functions but may play different roles within the constraints of their gene family, such as differential targeting as seen in some inhibitors of NF-κBs [45] or differential expression as seen in some PTPs [46]. Alternatively, conserved function across duplicated genes may be important for increasing the level of expression of functional classes of genes [26,51]. Despite the large proportion of genes under purifying selection, 7 genes appear to be evolving neutrally or under diversifying selection, potentially allowing a limited set of genes to acquire new functions or adapt to changes in host defenses.

The inter-segmental regions which separate the proviral genome segments are not predicted to contain protein coding genes. However, regions that map outside of proviral locus 1 are predicted to contain 9 genes, and potential function has been assigned to some of them, e.g., N-myristoyltransferase, ecto-5'-nucleotidase, and hyaluronidase (Figure 1). It is interesting to note that viral proteins are often modified with a lipid tail, hyaluronidase is a component of venom [52] that hydrolyzes complex carbohydrate structures allowing tissue diffusion, and ecto-5'-nucleotidase is involved in the extracellular formation of adenosine, a regulator of innate immune responses [53,54]. It is unclear whether these regions constitute part of the GiBV proviral genome but there is a striking difference in the structural complexity of these predicted gene models and those present in proviral locus 1. Nevertheless, it is tempting to speculate that proteins encoded in the flanking regions, perhaps as components of ovarian fluids, and genes that are located close to other proviral segment loci, may play a role in GiBV biology. Also notable is a sex-linked wasp gene coding for a homolog of female-lethal(2) [*fl(2)d*] that is present in region I. In *Drosophila fl(2)d *plays a critical role in alternative splicing regulation of genes involved in sex determination (including *Sex-lethal *and *transformer*), dosage compensation, oogenesis, and differentiation, as well as non sex-specific functions, and is expressed throughout larval and adult life [55-58]. Since excision of proviral genome segments from the wasp chromosome and encapsidation into virion particles occurs only in females, it is possible that regulation of this sex-linked process is related, at least in part, to expression of *fl(2)d*.

### A proviral genome segment excision motif is highly conserved across bracoviruses

The presence of a near perfect AGCTTT direct DNA sequence repeat was discovered at the boundaries of proviral genome segment sequences and flanking sequences (Figure 3). As the viral genome segment sequences contain a single copy of this repeat, it appears to define the site of proviral genome segment excision. This suggests an excision mechanism via conservative site specific recombination as described for formation of other PDV genome segments [12,33,39]. The presence of two SNPs within this repeat at the junction of proviral segment sequences and the ability to follow these nucleotide differences from the proviral to the viral genome segments suggests that the site of proviral genome segment excision and circularization must be located between the first and fifth position within the AGCTTT repeat (Figure 3). A study of excision sites in CiBV similarly concluded that GCT was the preferred site of excision [40].

An extended but different sequence motif around the excision site was identified at the 5' and 3' proviral genome segment junction sequences using MEME and the recombined sequence motif is found on viral genome segment sequences (Figure 3). While sequence conservation exists on both sides of excision sites, a higher level of conservation is seen in the side of the motif which is retained in circularized segment, and in particular at the 5' junction. The asymmetry of the 5' and 3' sequence motifs suggests that there is directionality to the recognition of excision sites. Since recombined sites have a different motif we predict they are no longer substrates for the excision enzymes. Excision and circularization of segments from a large precursor molecule could occur via release of single segments or a smaller molecule containing multiple segments. In the latter case the segments flanking the site of circularization would no longer be available for excision. For example, if a molecule encompassing 1p through 3p in proviral locus 1 were excised, only 2p would remain a substrate for subsequent excision and circularization (Figure 1). Such a pathway could contribute to differences in the abundance of packaged viral genome segments but it portrays a complex scenario. Assuming that sequence coverage of a viral genome segment in our shotgun sequencing approach correlates with the abundance of the segment it is interesting to note that the GiBV viral genome segments encoded in proviral locus 1 appear to be present in about the same levels (Table 4), suggesting that generation of intermediate excision products is not a common occurrence. The sequencing data also suggest that intermediates or by-products of excision, if they occur, are excluded from the packaging process, perhaps by the presence of inter-segmental DNA.

We found that the predicted site of excision/circularization and the recombined extended motif present in GiBV viral genome segments is also present in CcBV and MdBV viral genome segments (Figure 3). Conservation of the GCT portion of the excision repeat sequence exists in the CiBV viral genome segment sequences that are available [40], although more CiBV sequences will be required to determine how closely the CiBV extended motif mirrors that of GiBV, CcBV, and MdBV. As *C. congregata*, *G. indiensis*, and *M. demolitor *are all members of Microgasterinae, the most derived clade of bracovirus-bearing braconids, and *C. inanitus *is a member of Cheloninae, the most basal clade of the bracovirus-bearing wasps [59,60], it is possible that the predicted excision motif is one of the very few sequence features that is highly conserved across bracoviruses, and provides additional support for the hypothesis that bracoviruses have a single evolutionary origin [20,60]. This observation also predicts conservation of the enzyme(s) involved in BV proviral genome segment excision and circularization.

### Selective pressure on non-coding DNA in proviral segment sequences in locus 1

Analysis of SNP data derived from sequencing the GiBV viral genome from an outbred population of female wasps revealed that non-coding sites in the 8 viral genome segments derived from locus 1 had a significantly lower SNP density than synonymous sites within coding DNA. As we presume synonymous sites to be evolving neutrally, this result suggest that there is likely to be selective pressure on non-coding DNA. The lack of difference between rates of change at non-coding and non-synonymous sites suggests that in these segment sequences, non-coding DNA may be as highly conserved as coding DNA. Such areas could encode non-coding RNAs or contain sequence motifs vital to DNA replication, gene expression or segment packaging. Limited experimental evidence support the idea that PDV non-coding DNA is functional–studies of CsIV segment B found 2 sequences of 0.6 and 1.2 kbp which are transcribed but do not encode proteins [61,62].

### Proviral locus 1–a genetic island?

Several differences between the cluster of 8 GiBV proviral segment sequences which are excised and packed into virus particles and flanking DNA suggest that proviral segment sequences are not simply host genetic elements evolved for the export of wasp parasitism genes. For example, the proviral segments exhibit similar nucleotide compositions to each other but their G+C composition and dinucleotide frequencies differ from those of inter-segmental regions and flanking regions I-IV (Table 2 and Figure 2). Given the estimated age of the integration of bracoviruses into the wasp genome, ~74 million years, and using substitution rates estimated from *Drosophila *[63], one would predict that a sufficient period of time has passed for the process of ameliorization, i.e., the adjustment over time of the nucleotide composition of the integrated DNA to that of the resident genome [64], to have occurred. The different nucleotide composition of the proviral segment sequences may be maintained or its ameliorization may be slowed by the purifying selection found to be acting on both non-coding and coding DNA. However, as differences in nucleotide composition can be caused by different origins of DNA [64] or by the widespread purifying selection itself [65], the origins of the compositional differences between proviral and flanking DNA remain to be determined. Additionally, it is possible that inter-segmental and flanking regions, rather than the proviral segment sequences, differ from the remainder of the wasp genome.

## Conclusion

Here we provide the first report of a 223 kbp region of genomic DNA from the braconid *Glyptapanteles indiensis*, and the characterization of a cluster of 8 proviral genome segments encoded within it. Our data show that, contrary to current concepts of bracovirus proviral genome organization, the proviral segments are not entirely contained within a single tandem array in the wasp genome. However, it remains unclear whether the multiple GiBV proviral loci are linked on a single wasp chromosome as a macrolocus, and how representative this pattern is of BVs as a whole. The dispersed nature of GiBV proviral genome segments raises the question as to how to define proviral DNA within the wasp genome. It is reasonable to propose that sequences which can be shown to be physically linked to proviral genome segment sequences within amplified precursor molecules should be classified as part of the proviral genome. Whether such studies will reveal the entire composition of a proviral genome remains to be determined, as it is not known whether all genes involved in virion formation are components of precursor molecules.

Our study provides, for the first time, evidence for widespread purifying selection acting on BV non-coding DNA, suggesting that a large amount of the non-coding DNA in bracoviral genomes may be functional. Our analysis also reveals a variety of notable differences between flanking and proviral genome segment sequences. We hypothesize that selection acting on proviral DNA is maintaining the distinctive nucleotide composition of the proviral genome. However, the origins of these differences remain unknown. Neither proviral locus 1 nor any of the BV viral genomes sequenced to date encode homologs of known viral coat proteins or components of a transcription or DNA replication machinery, which are often the only genes conserved enough for viral phylogenetic studies. Identification of genes that perform these functions in *Glyptapanteles indiensis *will be essential for determining whether GiBV has a viral or cellular origin. As multiple lines of evidence, including the conserved excision motif described herein, support the hypothesis of a single evolutionary origin of BVs, an understanding of the evolutionary history of GiBV will reveal much about the evolution of BVs as a whole.

## Methods

### Rearing of parasitoid wasps

Outbred populations of *Glyptapanteles indiensis*, solitary endoparasitoids of gypsy moths (*Lymantria dispar*), were maintained at the USDA-ARS-Beneficial Insects Introduction Research Unit, Newark, Delaware, as part of a biocontrol program. The colony was founded in May 1998 from a shipment of 168 moths collected from 4 localities in India. In May 2002, the colony was outcrossed with 242 moths collected from the same localities. The mean colony size was 400 with an average sex ratio of 7 females:13 males. Host larvae were fed on a high wheat-germ diet. Both wasp and host larvae were maintained at 26°C, 58% relative humidity, and a light-dark (L:D) cycle of 16L:8D hr according to established protocol [66]. *G. indiensis *parasitize late first instar gypsy moth larvae. Cocoons formed from parasitized hosts were stored at 24°C until adult parasitoid emergence and then separated by sex. *G. indiensis *larvae were dissected from parasitized host 10 days post parasitization, briefly rinsed in phosphate buffered saline (PBS), flash frozen in liquid nitrogen and stored frozen at -80°C.

### Virion purification and DNA extraction

Virions were purified from *G. indiensis *females using established protocols [67]. Briefly, female wasps were anaesthetized in 75% ethanol and rinsed in PBS. Ovaries were dissected from the females in a drop of PBS and ruptured, draining the calyx fluid. Pooled calyx fluid was subsequently filtered through a 0.45 μm filter to remove eggs and cellular debris [68]. Viral DNA was extracted according to established protocol [41]. Briefly, viral DNA was isolated from the calyx fluid using a proteinase K/SDS buffer, DNA was extracted with phenol, precipitated with ethanol, and recovered by centrifugation.

### Identification of BAC clones containing proviral DNA

A BAC library of *G. indiensis *with a 120 kb average insert size was constructed by Amplicon Express [69], using a partial *Bam*HI digest inserted into an *Mbo*I site of a pECBAC1 vector. A nylon filter arrayed with 9,216 BAC clones was created from the library. In order to identify BAC clones containing proviral DNA, GiBV viral DNA was radioactively labeled with ^32^P-labeled α-dCTP (NEN/Perkin-Elmer) using the Redi-prime II DNA labeling kit (Amersham Biosciences). Labeled DNA was then purified using a QIAquick PCR purification kit (Qiagen). The filter was pre-hybridized at 65° for at least 3 hours with Rapid-hyb Buffer (Amersham Biosciences) and 500 μg of salmon testes DNA (denatured at 100°C, Sigma-Aldritch). The probe was added and allowed to hybridize overnight at 65°C. The filter was then washed 2 times for 60 minutes each at 65°C with a 0.1 × SSC/0.1% SDS solution, wrapped in plastic wrap, and autoradiographed using Kodac BioMax MS film.

### BAC DNA preparation and fingerprinting

BAC clones were grown in 5 mL LB with 12.5 μg/ml chloramphenicol overnight at 37°C and shaking at 200 rpm. BAC DNA was extracted using the Sigma Phaseprep BAC DNA Kit (Sigma-Aldritch) without the endotoxin removal step. BAC DNA was digested with *EcoR*I (Invitrogen) in a 1:150 dilution of RNase cocktail (Sigma Phaseprep Kit) at 37°C for 2 hours. Digested DNA was run overnight on a 1.2% agarose gel, stained with Vistra Green and imaged using a FluorImager SI (Amersham Biosciences). Gel images were processed using Image [70], and contigs were assembled using FingerPrintContig [71] using the default e-value of e-10.

### GiBV and BAC clone sequencing

Approximately 7.5 μg of GiBV DNA was sheared and DNA fragments in the size range of 3.5–4.5 kbp purified after separation by agarose gel electrophoresis. The fragments were blunt ended and, after addition of *Bst*XI adaptors, cloned into the *Bst*XI site of pHOS2. Shotgun libraries were made from the 2 BAC clones as described for GiBV DNA. Celera Assembler [72] and TIGR Assembler [73] were used to assemble random sequence data from the viral whole genome shotgun and BAC clones, respectively. Gap closure was assisted by a closure editor tool called Cloe that also permits the manual inspection and editing of sequence data. A variety of methods were used to close gaps including re-sequencing the ends of random clones, transposon assisted sequencing (GPS, New England Biolabs™) or "micro-library" construction of single or pooled templates, and conversion of physical gaps to sequence gaps using "POMP" (pipette optimal multiplex PCR) [74] and or/a "Genome Walker" kit (Invitrogen™).

### GiBV segment-specific PCRs

Primers were developed to be specific to 19 GiBV viral genome segment sequences. Primers were designed to be 22–26 nt in length, have a Tm of 62–65°C, a GC clamp, and a maximum identity to the remainder of the unclosed GiBV genome of 70%. Designed primers were tested for potential secondary structure using NetPrimer [75]. PCR was performed in a 10 μl solution which included 0.1 μl template DNA, 0.3 μl 50 mM MgCl_2_, 1 μl 10 × PCR buffer, 0.2 μ10 mM dNTPs, 7.9 μl H_2_O, 0.1 μl Platinum Taq (Invitrogen), 0.2 μl F primer (20 pm/μl), and 0.2 μl RC primer (20 pm/μl). PCR protocol was 94° for 2 min; 35 cycles of 94° for 30 sec, 58° for 30 sec, 72° for 45 sec; followed by 72° for 7 min.

### Derivation of consensus GiBV segment sequences

As shotgun sequencing of the GiBV DNA was carried out using a sample pooled from a population of ~400 wasps, a large number of SNPs and indels were present in the sequence assembly. Because individual sequence reads could not be associated with individual wasps, a conical consensus sequence was generated for each viral genome segment using the SliceTools package [76]. At a given position in a conical consensus, all bases with a cumulative quality value within 50% of the highest cumulative quality value are assigned to that position.

### Annotation

Gene models were generated with a variety of software: Softberry's FGENESH [77] using both the honey bee (*Apis mellifera*) and fruit fly (*Drosophila melanogaster*) training sets, the Beijing Genome Institute's BGF [78] trained on the silkmoth (*Bombyx mori*), and GENSCAN [79] using the vertebrate training set. Predicted gene models were compared to gene models generated using cDNA from 2 gene families for their ability to predict correct intron-exon structure. Most of the gene finders accurately predicted the 2 intron structure of the p494 genes, with the exception of GENSCAN which predicted an extra exon. The single intron in p325 genes were significantly more difficult to predict – only FGENESH (*A. mellifera*) and BGF properly predicted these genes. FGENESH (*D. melanogaster*) and GENSCAN both mis-predicted the majority of intron-exon boundaries and showed a tendency to combine multiple genes into single genes with a large number of introns. Based on these results, a combination of FGENESH (*A. mellifera*) and BGF was used for gene prediction, in addition to the AAT package [43] which allows spliced alignment of proteins to genomic DNA thereby revealing potential exon-intron boundaries. Gene models from FGENESH were generally accepted except when multiple other sources of information contradicted those models. SignalP [80,81], TM-HMM [82], and GPI-SOM [83], were used to predict signal peptides, transmembrane domains, and glycosylphosphatidylinisotol anchors, respectively. Predicted genes were clustered into gene families using previously described methods [84], which utilize Pfam [85] and TIGRFAM [86] domains and calculate novel shared domains within the genome. Predicted GiBV proviral segment genes were analyzed for potential homology to genes in CcBV and MdBV and CsIV using BLASTP (CcBV only) and TBLASTN (CcBV and MdBV), with a cutoff of E = e-10.

### Nucleotide composition analysis

Relative dinucleotide frequencies [87], were calculated for each region > 500 bp in length except the flanking repeats, as they are expected to have highly biased dinucleotide frequencies. A Euclidean distance matrix between the regions was constructed from these frequencies. Regions were then clustered using the Neighbor-joining algorithm in PAUP* [88] and the resulting tree was visualized using PHY·FI [89].

### Motif analysis

Boundaries between the proviral segments and inter-segmental regions, and the inter-segmental regions themselves were analyzed for motifs using MEME [90]. In the first analysis a 103 bp DNA sequence (50 bp upstream to 50 bp downstream of the GCT excision motif) was extracted from each segmental boundary. The boundaries of proviral segments 1p, 3p, and 5 bp were reverse complemented so that orientation of the excision motif was the same for all sequences. All 16 sequences were analyzed together, and then split into 8 5' (upstream) and 8 3' (downstream) motifs relative to the directionality of the excision motif. Next, an analysis was conducted using the entire length of the 7 inter-segmental regions. Analyses used a minimal and maximal motif length of 5 and 100 bp, respectively. MEME was also used to search 30 CcBV [Genbank :AJ632304–AJ632333], 15 MdBV [Genbank:AY887894, AY875680–AY875690, AY848690, AY842013, DQ000240], and 5 CiBV viral genome segments [Genbank :AJ627175, AJ278677, AJ319654, Z58828, Z31378] for common motifs. All motifs were visualized using WebLogo [91,92].

### SNP analysis

Ambiguous consensus sequences were generated from the viral genome sequence by recalling contigs so that all high quality (quality value > = 30) base calls in the reads were represented in the new consensus as ambiguity codes. This ensured all variants of a given circle were encoded within a single consensus sequence, while preventing low quality sequencing error from introducing artificial polymorphisms. Then, the ambiguous viral genome segment consensus sequences were globally aligned to their corresponding proviral genome segment sequences using nucmer from the MUMmer package [93]. This alignment was parsed to determine the positions of all polymorphisms relative to the reference proviral sequence, including both substitutions and indels. Substitutions were found by mismatches in the alignment between the viral consensus sequence and proviral reference sequence. The distribution of polymorphisms was analyzed using the gene-snps tool from the AMOS package [94]. The tool examines each polymorphism to determine if it occurs within an exon, and if so, whether the change is synonymous or non-synonymous. Additionally, the tool estimates dN/dS for each gene using the unweighted pathway method [95]. The final analysis the tool performs is a test of independence between SNP density and sequence coverage (i.e. if more sequences covering any given position means that position is more likely to contain a polymorphism). To do so, it computes the Pearson's correlation of the polymorphism rate and depth of coverage using a sliding window of size 500 bp offset by 250 bp across each circle. Statistical significance of correlation coefficients were evaluated using a 2-tailed t test, where degrees of freedom equals the number of SNPs minus two. Differences between the relative number of substitutions of non-coding, synonymous, and non-synonymous sites were evaluated using Pearson's χ^2 ^test.

## Abbreviations

PDV, polydnavirus; BV, bracovirus; IV, ichnovirus; GiBV, *Glyptapanteles indiensis *bracovirus; CcBV, *Cotesia congregata *bracovirus; MdBV, *Microplitis demolitor *bracovirus; CiBV, *Chelonus inanitus *bracovirus; CsIV, *Campoletis sonorensis *ichnovirus; SNP, single nucleotide polymorphism; dN/dS, ratio of non-synonymous to synonymous substitutions; PTP, protein tyrosine phosphatase

## Authors' contributions

VN and DEGR conceived the project. VN, CAD, and DEGR coordinated the project. CAD, DEGR, VN, and MJP designed and performed laboratory procedures and experiments. CAD, MCS, and VN designed and performed computational analyses. CAD, VN, and DEGR wrote the manuscript. CAD and BJH participated in annotation. JBH and LJT participated in genome closure. RWF reared parasitoids. DWF, BST, and DC participated in library construction. All authors read and approved this manuscript.
